# Comparison of Different Response Time Outlier Exclusion Methods: A Simulation Study

**DOI:** 10.3389/fpsyg.2021.675558

**Published:** 2021-06-14

**Authors:** Alexander Berger, Markus Kiefer

**Affiliations:** Section for Cognitive Electrophysiology, Department of Psychiatry, Ulm University, Ulm, Germany

**Keywords:** response time, reaction time, outlier exclusion, simulation study, mental chronometry

## Abstract

In response time (RT) research, RT outliers are typically excluded from statistical analysis to improve the signal-to-noise ratio. Nevertheless, there exist several methods for outlier exclusion. This poses the question, how these methods differ with respect to recovering the uncontaminated RT distribution. In the present simulation study, two RT distributions with a given population difference were simulated in each iteration. RTs were replaced by outliers following two different approaches. The first approach generated outliers at the tails of the distribution, the second one inserted outliers overlapping with the genuine RT distribution. We applied ten different outlier exclusion methods and tested, how many pairs of distributions significantly differed. Outlier exclusion methods were compared in terms of bias. Bias was defined as the deviation of the proportion of significant differences after outlier exclusion from the proportion of significant differences in the uncontaminated samples (before introducing outliers). Our results showed large differences in bias between the exclusion methods. Some methods showed a high rate of Type-I errors and should therefore clearly not be used. Overall, our results showed that applying an exclusion method based on z-scores / standard deviations introduced only small biases, while the absence of outlier exclusion showed the largest absolute bias.

## Introduction

Response time is the variable of interest in a large number of studies in (cognitive) psychology (Donders, 1969; Posner, 1978; Luce, 1991). Response times are collected in tasks stressing the subjects to respond as fast and as accurate as possible. Therefore, they are measures of maximum performance equally weighting response speed and accuracy. Nevertheless, performance varies across trials of such tasks and the response time of some trials typically strongly differ from the response time distribution. Such trials / response times are called *outliers* (Cousineau and Chartier, 2010) and are usually excluded from data analysis. However, there exist several methods for excluding outliers with different assumptions and formulas. The aim of this article is to compare different methods for excluding outliers and to investigate how these methods perform with respect to recovering the uncontaminated response time distribution. Before describing response time outliers more precisely, some expressions that will be used in the present article should be specified. The terms response time and reaction time (RT) are both typically used for describing the response latency in (psychological) studies. In this article, the terms will not be distinguished. We will refer to the RT distribution not containing outliers as “valid,” “genuine,” or “uncontaminated” RTs. For the RT distribution distorted by outliers, the term “contaminated” RT distribution will be used. Furthermore, we only focus on methods, which exclude outliers, but do not discuss methods which correct outlying RTs by transforming the contaminated RT distribution or by replacing them.

To clarify, which observations are outliers, there are two major questions: I. What is the probability of observing such an (extreme) RT (Ratcliff, 1993; Motulsky and Brown, 2006; Leys et al., 2013)? II. What is the underlying (cognitive) process leading to the occurrence of such a RT (Luce, 1991; Ratcliff, 1993; Cousineau and Chartier, 2010)? As one will see, both questions overlap to a certain degree, and outlier exclusion methods differ in how they address these issues. Outliers can be divided into short and long outliers (Ratcliff, 1993), i.e., outliers at the left and right tail of the RT distribution (also labeled as fast and slow outliers). For the underlying process leading to short outliers, a response without appropriate processing of the stimulus is discussed. Short outliers are therefore also referred to as “fast guesses” (Whelan, 2008; Cousineau and Chartier, 2010). In two-alternative forced choice tasks, the mean accuracy of short outliers should therefore be around 0.5 as a consequence of guessing. In contrast, possible underlying processes for the occurrence of long outliers are lack of attention or distraction (Whelan, 2008; Cousineau and Chartier, 2010). As such processes are certainly not of interest, outliers are removed to ensure that the analyzed RT distribution only consists of data generated by the (cognitive) processes intended to be examined^1^. Excluding outliers should therefore increase the signal-to-noise ratio (given that the proportion of correctly excluded outliers is larger than the proportion of incorrectly excluded valid RTs). To sum up, in view of question II, short and long outliers are thought to be based on different processes as the genuine RT distribution. Note at this point, that although the processes underlying short and long outliers may differ, the methods for excluding outliers treat them in a comparable fashion and are mainly based on the probability to observe such a RT (question I; see section “Outlier Exclusion Methods”). RTs derived from different processes that are not at the tails of the RT distribution (no short and long outliers) cannot be detected by outlier exclusion methods, which exclude outliers based on an upper and lower threshold (Whelan, 2008). Consequently, following question I, for such methods it is only possible to account for outliers at the tails of the RT distribution. As it is not possible to account for outliers “inside” the RT distribution, question II can only be reasonably applied to outliers at the tails. When applying an exclusion method based on an upper and lower threshold, one has to stay with the assumption that all RTs inside the distribution emerge from the same processes. Therefore, question II is naturally restricted to outliers, which can be detected by means of question I. As almost any outlier exclusion method is calculated by means of the RT distribution, several candidates for RT distributions should be mentioned first.

The typical RT distribution shows a bell-like shape with a tail at the right side. Valid RTs, i.e., RTs generated by the process of interest, should not start before 100–200 ms (Luce, 1991; Whelan, 2008), because there is at least the need to encode the stimulus and execute the response (Ashby and Townsend, 1980). Therefore, valid RTs are physiologically limited to a minimum boundary at the left side of the distribution. However, there is a debate which distribution provides the best fit to empirical observed RTs.

As empirical RT distributions are typically skewed, they should not be described by a Normal (Gaussian) distribution (Ratcliff, 1979; Whelan, 2008; Cousineau and Chartier, 2010). As an alternative, the Gamma distribution was discussed for modeling RTs (McGill and Gibbon, 1965). This distribution assumes a series of different, discrete stages, which the cognitive system processes one after the other. It theoretical focus rests on sensory processing in psychophysics (McGill and Gibbon, 1965) and may therefore not be suitable to account for the broad range of (cognitive) processes involved in RT tasks. A further candidate is the Wald distribution (Luce, 1991). It describes the density of a diffusion process to a single boundary (Matzke and Wagenmakers, 2009). This renders it unclear how the Wald distribution can account for more complex tasks with two (or more) responses (boundaries) (Matzke and Wagenmakers, 2009). A widely used approach to describe RT distributions is the Ex-Gaussian distribution (Ratcliff, 1979; Whelan, 2008; Marmolejo-Ramos et al., 2015; Moret-Tatay et al., 2018). It is composed of the sum of a Gaussian (Normal) and an Exponential distribution. Despite there is a lack of detailed theoretical derivation, it fits empirical RT distributions very well (Ratcliff, 1979; Whelan, 2008; Marmolejo-Ramos et al., 2015). The distribution consists of three parameters: μ (mean of the Gaussian distribution), σ (standard deviation of the Gaussian distribution) and τ (decay rate of the Exponential distribution). Considering the interpretation of these parameters, recent research showed that they cannot be uniquely mapped to distinct processes (e.g., Matzke and Wagenmakers, 2009; Rieger and Miller, 2020). The Gaussian part of the distribution causes the “bell-shape,” while the Exponential part causes the tail at the right side of the distribution. As these distributions themselves do not take outliers into account, common methods for outlier exclusion assess, how a single RT deviates from the RT distribution. The more a RT deviates from the majority of RTs, the more likely it is an outlier. A set of outlier exclusion methods based on RT distributions will be discussed in the following section in detail.

The methods for outlier exclusion, which will be examined in this simulation study, are introduced in the following sections. First, one option to treat RT outliers is to not treat them at all, i.e., analyzing the data without excluding outliers. This method may only be appropriate if one can be convinced that the same (cognitive) processes may generate the entire observed RT distribution (compare question II). However, the theoretical and methodical prerequisites of this assumption should be extensively taken into account beforehand, rendering it hard to draw such a conclusion. Instead, if one could be sure that outlier influences are equal across experimental conditions, it may be appropriate to not correct for outliers, as their influence is negligible when comparing experimental conditions. Nevertheless, as for the underlying (cognitive) processes, it is questionable how one can (reliably) specify the influence of outliers in observed RT data.

One possibility to correct for RT outliers is to exclude outliers above or below *a priori* determined absolute cutoffs. This method is often called truncation or trimming (Bush et al., 1993; Ratcliff, 1993; Ulrich and Miller, 1994). As these terms are sometimes also used for outlier exclusion methods based on relative cutoffs, we remain to refer to this method as outlier exclusion based on (absolute) cutoffs. The rationale to use absolute cutoff values is simple: as there is a minimum RT for meaningful responses (Ratcliff, 1993; Whelan, 2008), “fast guesses” can be eliminated by excluding all RTs below this criterion. For slow outliers, RTs beyond a threshold of e.g., 2 s should clearly be no performance at maximum and consequently excluded. Nevertheless, defining the absolute cutoff values is not trivial, therefore requiring informed a-priori knowledge about the overall response speed in a given task. Furthermore, it is not clear if all outliers fall outside the cutoff boundaries and if all valid RTs fall within these boundaries (Ulrich and Miller, 1994). This problem cannot be solved by exclusion methods based on relative cutoffs as well. However, these methods provide a standardized approach to determine the cutoff values.

There are several outlier exclusion methods based on relative cutoffs. For the present simulation study, we will focus on cutoffs based on the mean ±2 or 3 standard deviations (SDs) and the Tukey outlier exclusion method (Tukey, 1977). These methods have in common that the cutoffs are calculated using descriptive statistics of the observed RT distribution. A widely used method (see Leys et al., 2013) is to exclude RTs which are larger/smaller than the mean ±2 SDs (Miller, 1991; Zimmerman and Williams, 2000). About 95% percent of values from a Normal distribution lie within the mean ±2 SDs (although empirical RT distribution are clearly not normal distributed). Consequently, this method should exclude a similar proportion of RTs when applied to different data sets (with similar distribution). Considering methods based on the mean and SD, there is a variety of cutoffs used in RT research (cf. Miller, 1991). To compare different thresholds, we also tested a method based on the mean ±3 SDs, which should exclude about 0.5% of data points given a Normal distribution. Note that the maximum possible z score in a sample depends on the sample size (Shiffler, 1988). Using a criterion based on the mean ±3 SDs (*z* = 3), the sample size must be >10 to possibly detect any outlier. Given that RT distributions are skewed, critics for these methods mention a different likelihood of exclusion for fast and slow RTs (Miller, 1991). Furthermore, as outlier influence mean and SD, the outlier exclusion criterion is influenced by the (magnitude of) outliers itself (Miller, 1991). Tukey (1977) provided another method for outlier exclusion based on relative cutoffs, which is often referred to as *Tukey’s method*. It is the criterion used in visual outlier detection using boxplots (Dawson, 2011) and is calculated with means of the interquartile range (IQR). The IQR is defined as the difference of the third quartile (0.75 quantile) minus the first quartile (0.25 quantile) of a distribution. Quartiles are robust against outliers at the tails (in large samples). Tukey’s method identifies RTs as outlier, which are larger than the third quartile plus 1.5 times the IQR (> *q*_0.75_ + 1.5×*IQR*) or smaller than the first quartile minus 1.5 times the IQR (< *q*_0.25_−1.5×*IQR*). How conservative this method is (how many RTs are excluded as outliers) is strongly influenced by the sample size (Dawson, 2011). Similar to the idea of a criterion based on SDs and Tukey’s method, we will also test in the present simulation study two methods directly based on quantiles. For example, by excluding RTs smaller than the 0.025 quantile and larger than the 0.975 quantile, the proportion of excluded RTs can be exactly set to 5%. As a consequence of excluding fixed proportions, these methods will always exclude valid RTs if no outliers are present in the data (Ulrich and Miller, 1994).

A recent paper strongly suggested to use the Median Absolute Deviation (MAD) method for outlier exclusion (Leys et al., 2013). The authors regarded this method as especially robust, as the median is not influenced by outliers and the exclusion criterion is therefore not affected by outliers (Leys et al., 2013). Note that other authors argued to not use the median for describing RT data, as sample medians overestimate population medians in small skewed samples (Miller, 1988). The MAD is defined as: *MAD* = *b*×*Med*(|*x*_*i*_−*Med*(*x*)|). *b* is a constant (usually b = 1.4826; based on the assumption of a normal distribution; see also Rousseeuw and Croux, 1993). This constant is multiplied with the median (*Med*) of the absolute value (| |) of all observations minus the median of all observations (*Med(x)*). To sum up, the MAD consists of the median of absolute deviations from the (sample) median times a constant. RTs are excluded as outliers if they exceed the median ±2.5 × MAD (Leys et al., 2013, suggested a threshold of 2.5 as default).

A recent review of outlier detection and treatment suggested an outlier exclusion method for RT data based on a transformation approach (Cousineau and Chartier, 2010). The rationale is to achieve a symmetrical shape for the RT distribution after transformation, resulting in the same exclusion probability for long and short outliers. Following formula is used for the transformation: $y_{i}=\sqrt{\frac{x_{i}-\text{min}\,\left(x\right)}{\max\left(x\right)-\text{min}\,\left(x\right)}}$

For each transformed value, the square root of the untransformed value minus the minimum value of the sample divided through the sample range is calculated. The fraction bounds all values between 0 and 1, while the square root enlarges small values (Cousineau and Chartier, 2010). Afterwards, these values are z-transformed and values exceeding a particular z-score (e.g., 2 or 3) are excluded. For the present simulations, we excluded RTs associated with a z-score larger/smaller than ±2 as outliers.

In view of the different approaches these procedures use, the aim of this study is to compare different RT outlier exclusion methods using simulations. Several studies using simulations already investigated the influence of outliers on RTs including methods to correct for this influence. In the following section, we will outline which research questions these studies addressed, leading to the questions, which the present study aims to clarify. The influence of outlier exclusion on RT distribution is generally recognized. Nevertheless, there is no consensus whether the influence on the RT distribution due to the presence of outliers or due to bias introduced by outlier exclusion is more adverse. Cousineau and Chartier (2010) argued to use a conservative threshold to exclude outliers, i.e., keeping the original data with a high probability. Using such a criterion, the probability to erroneously exclude valid RTs is low. Outlier exclusion should also be taken with care when comparing conditions with different sample sizes (Miller, 1991). In such a situation, the outlier exclusion can introduce an asymmetric bias. Ratcliff (1993) investigated several outlier exclusion methods as well as the influence of outliers on the RT distribution. The results showed a reduction of power to detect a mean difference when outliers were present. Outlier exclusion could compensate for this reduction. Furthermore, outlier exclusion did not increase the Type-I error rate. In contrast to Cousineau and Chartier (2010), a lower cutoff threshold for slow outliers (associated with a higher proportion of excluded RTs) was associated with a lower reduction in power (Ratcliff, 1993). Overall, Ratcliff suggested to exclude outliers based on specific absolute cutoff values, which should be derived according to characteristics of the RT distribution. Ulrich and Miller (1994) investigated the influence of outlier exclusion based on excluding observations according to a lower and upper cutoff in general. They systematically varied the proportion of excluded fast and slow RTs and investigated (among others) changes on summary statistics and statistical power. Their simulations showed a large bias on summary statistics, but a beneficial effect on statistical power. Appropriate exclusion cutoffs could compensate for the reduction of power due to outlier. The authors recommended in view of the large bias on summary statistics to not exclude outliers at all or to use conservative thresholds which exclude only a few RTs to ensure that the proportion of excluded valid RTs is very small (Ulrich and Miller, 1994). In view of the competing recommendations, i.e., to exclude outliers (Ratcliff, 1993) or to not exclude / exclude a tiny proportion at most (Ulrich and Miller, 1994; Cousineau and Chartier, 2010), the present study aims to further examine whether applying an outlier exclusion method introduces a larger bias compared to retaining the outliers and whether this bias depends on the applied outlier exclusion method. Therefore we compared several outlier exclusion methods with the absence of outlier exclusion, covering widely used methods (cf. Leys et al., 2013) as well as recently suggested approaches (Cousineau and Chartier, 2010; Leys et al., 2013). In contrast to earlier research (Ratcliff, 1993; Ulrich and Miller, 1994), which used fixed effect magnitudes for determining the influence of outlier exclusion on power, we varied the effect on a continuous scale, ranging from 0 to 100 ms. Influences of outliers as well as exclusion methods can be accordingly compared throughout a broad range of possible effects. This allows to investigate whether the bias of outlier exclusion as well as the bias of outliers (i.e., no exclusion of outliers) differ across the absence of any effect up to very large effects. The design of the present simulation study will be introduced in the following section.

To assess the influence of outlier exclusion, sets of two RT distributions with a given (population) difference were simulated, valid RTs were replaced by outliers, and different outlier exclusion methods were applied. Success of outlier exclusion was measured in terms of bias. Bias was defined as the deviation of the proportion of rejected Null hypotheses per exclusion method from the respective proportion in the valid samples.

To assess how successful different methods can exclude RT outliers, it is necessary to determine outliers. A possible approach are simulation studies, in which one can simulate genuine RT distributions and outliers by pre-defined parameters. Accordingly, this simulation study rests on three lists of data: (1) Valid RT distributions. (2) Contaminated RT distributions including outliers. (3) RT distributions with outliers excluded based on the different methods. Therefore, the influence of outliers can be assessed by comparing the 1st and the 2nd list, and the success of outlier exclusion can be assessed by comparing the 3rd and the 1st list. For details of the simulation process, see the section “Methods.” Instead of investigating single RT distributions, common RT research tests RTs in different conditions against each other. To account for such designs, we compared the outlier exclusion methods in terms of bias, i.e., how accurate they are in correctly rejecting or keeping the Null hypothesis. We simulated two RT distributions per simulation iteration, which were drawn from two populations. The population parameters were the same except of a *μ* difference between both conditions. These distributions were added to the 1st list (valid RT distributions). Subsequently, randomly chosen valid RTs were replaced with outliers (2nd list) and the contaminated distributions including outliers were corrected according to the several methods (3rd list). As every iteration consisted of a single RT distribution per condition (drawn from populations only differing in *μ*), we directly tested RT samples and not mean RTs. One iteration can therefore be seen as RT data from two samples representing two different conditions. Nevertheless, as we aggregated the results per one given population difference across a range of iterations (5000 iterations, see the section “Methods”) with randomly varying mean, skewness, outlier proportion and variability, the results should be generalizable to the broad range of available RT distribution characteristics in empirical research.

## Methods

**FIGURE 1:**
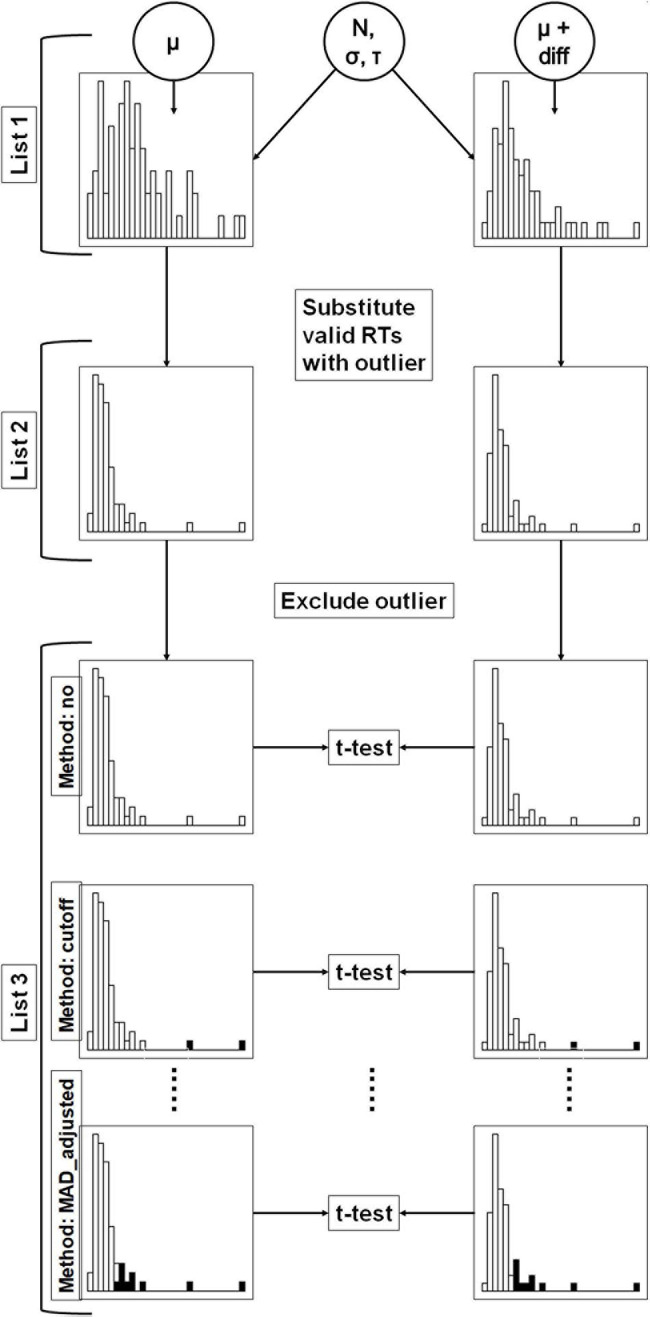
Flowchart of the present simulation study. In a first step (list 1), valid response time distributions were simulated in two conditions. The distributions in both conditions were simulated with equal parameters *N*, *σ* and *τ*. In the second condition, a constant (*diff*) was added to *μ* of the first condition. In the second step (list 2), randomly chosen valid RTs were replaced by outliers. In the third step (list 3), outliers were excluded according to the different outlier exclusion methods. The figure shows three methods as examples. Excluded response times are highlighted with black color. The resulting distributions were compared with *t*-tests.

The simulations and statistical analyses were performed using R, version 4.0.2 (R Core Team, 2020). Graphics were created with help of the package “ggplot2” (Wickham, 2016). Data was processed with the help of the package “data.table” (Dowle and Srinivasan, 2021). Scripts and simulated data were uploaded to the Open Science Framework^2^. Simulated data is thought to represent RTs in two different conditions. The (population) mean difference between both conditions was systematically varied. Afterwards, outliers replaced valid RTs according to two different approaches and were excluded using several methods. The proportion of significant *t*-tests was compared between the outlier exclusion methods. Figure 1 describes the design of the simulation approach used. Details are provided in the following sections.

### Simulating Response Time Distributions

We used Ex-Gaussian distributions to simulate RTs in accordance with earlier work (Miller, 1991; Ratcliff, 1993). Trial number (*N*), mean (*μ*), standard deviation (*σ*) and exponential decay rate (*τ^3^*) were simulated from Uniform distributions (*U*) using the following boundaries:

N∼U⁢(20, 100)

μ∼U⁢(250, 500)

σ∼U⁢(20, 50)

τ∼U⁢(150, 200)

The unit of the RT distributions (*μ*, *σ*, *τ*) was the millisecond. The boundaries of *N* were chosen to represent a broad range of available trial numbers in RT research. The boundaries of *μ*, *σ* and *τ* were chosen to capture the range of the chosen parameters in former studies simulating RT distributions (Miller, 1991; Ratcliff, 1993; Whelan, 2008). We did not vary the parameters of the Ex-Gaussian distribution in a factorial manner to ensure the generalizability of the results across a broad range of parameter combinations. *N*, *σ* and *τ* were held constant for each pair of simulated samples. Accordingly, each pair of samples can be thought of representing the RTs of two conditions drawn from the same distribution, the only parameter varying between conditions is *μ*. In condition 1, *μ* was drawn from the Uniform distribution described above. In condition 2, a fixed value was added to *μ* of condition 1, representing the (population) treatment effect. The fixed value (difference between both conditions) was varied from 0 to 100 ms in 1 ms steps, resulting in 101 possible differences. For each difference, 5000 pairs of samples were simulated, resulting into a total number of 101 × 5000 = 505,000 pairs of samples.

### Simulating Outliers

The simulation of outliers was adapted from a function in the HDDM tool (Wiecki et al., 2013). The number of outliers were simulated using a Uniform distribution:

n⁢(o⁢u⁢t⁢l⁢i⁢e⁢r)∼U⁢(0, 0.1×N)

*N* denotes the trial numbers. The resulting number was rounded to an integer. As a consequence of this approach, the samples included between 0% and 10% outliers. We restricted the upper boundary of outliers to 10%, as we would expect RT data with more than 10% of outliers to not represent “performance at maximum.” RT data with a higher amount of outliers should be excluded as a whole from the analysis. Outliers were classified into short and long outliers. The proportion of long outliers was simulated using a Beta distribution:

P(longoutlier)∼B(α=5,β=1)

This results in an expected value of an outlier being a long outlier of about 0.83. The larger probability for long outliers was thought to reflect the physiologically restricted smaller range of possible RTs for short outliers. The remaining outliers were short outliers: *P*(*shortoutlier*) = 1−*P*(*longoutlier*). The respective proportions for long and short outliers were multiplied with n(outlier) and rounded to integers, resulting in the number of short and long outliers. We tested two different approaches to simulate long and short outliers.

#### Approach 1 - *Tails*

Approach 1 was adopted from the HDDM tool (Wiecki et al., 2013) and simulated outliers at the tails of the distribution. Accordingly, long outliers were always larger than the maximum valid RT and short outliers were always smaller than the minimum valid RT. Such an approach is thought to represent a definition of outliers purely based on the probability to observe such RTs (compare question I in the Introduction). Accordingly, the definition of outliers is based on the same mechanisms as the outlier exclusion. The following formulas were used:

R⁢T⁢(l⁢o⁢n⁢g⁢o⁢u⁢t⁢l⁢i⁢e⁢r)=U⁢(0, 1)×2000⁢m⁢s+max⁢(R⁢T)R⁢T⁢(l⁢o⁢n⁢g⁢o⁢u⁢t⁢l⁢i⁢e⁢r)=U⁢(0, 1)×2000⁢m⁢s+max⁢(R⁢T)R⁢T⁢(s⁢h⁢o⁢r⁢t⁢o⁢u⁢t⁢l⁢i⁢e⁢r)=U⁢(0, 1)×(min⁡(R⁢T)-100⁢m⁢s)+100⁢m⁢sR⁢T⁢(s⁢h⁢o⁢r⁢t⁢o⁢u⁢t⁢l⁢i⁢e⁢r)=U⁢(0, 1)×(min⁡(R⁢T)-100⁢m⁢s)+100⁢m⁢s

*U* denotes a Uniform distribution, *min(RT)* and *max(RT)* the minimum and maximum of the sample RT (before introducing outliers).

#### Approach 2 - *Overlap*

Approach 2 was adopted from Ulrich and Miller (1994), who simulated fast and slow outliers independent of the valid RT distribution. Outliers could therefore overlap with the distribution of valid RTs. This approach can be therefore thought to represent an outlier definition based on different cognitive processes independent of the genuine RT distribution (compare question II in the Introduction). In contrast to Ulrich and Miller (1994), who used a fixed *μ* for the generation of outliers, we calculated this parameter depending on the characteristics of the valid RT distribution. This was a consequence of valid RTs simulated with not fixed, but varying parameters. Ex-Gaussian distributions were used for the simulation of outliers in accordance to the simulation of valid RTs. The following formulas were used:

RT(longoutlier)=ExGaussian(μ=mean(validRTs)+700ms,R⁢T⁢(l⁢o⁢n⁢g⁢o⁢u⁢t⁢l⁢i⁢e⁢r)=ExGaussian(μ=mean(validRTs)+700ms,

σ=U⁢(20⁢m⁢s, 50⁢m⁢s)+10⁢m⁢s,σ=U⁢(20⁢m⁢s, 50⁢m⁢s)+10⁢m⁢s,

τ=U(150ms, 200ms))τ=U(150ms, 200ms))

RT(shortoutlier)=ExGaussian(μ=mean(validRTs)-200ms,R⁢T⁢(s⁢h⁢o⁢r⁢t⁢o⁢u⁢t⁢l⁢i⁢e⁢r)=ExGaussian(μ=mean(validRTs)-200ms,

σ=20⁢m⁢s,σ=20⁢m⁢s,

τ=10ms)τ=10ms)

*ExGaussian* denotes an Ex-Gaussian distribution, *U* a Uniform distribution. *μ* was calculated depending on the mean of the distribution of valid RTs. The values of 700 ms and 200 ms were chosen to ensure the same *μ* difference between valid RTs and long and short outliers as in Ulrich and Miller (1994). *σ* for long outliers was based on the possible range of *σ* for valid RTs + 10 ms, to ensure broader variability in the processes underlying long outliers. *σ* for short outliers was set constant due to the physiological limited variability in short outliers (“fast guesses”). *τ* for long outliers was simulated in accordance with valid RTs, while it was fixed to a small value for short outliers to achieve a more symmetric distribution. An exponential tail is not plausible in small outliers based on guessing. If the generation of (short) outliers resulted in a negative RT value, it was multiplied with -1.

In both approaches, outliers were simulated separately in the two conditions, but the number of outliers was fixed across conditions. Randomly chosen valid RTs were replaced by outliers. Consequently, sample size did not differ between the valid and respective contaminated RT distributions. Throughout the article, we will refer to approach 1 as approach “*tails*” and to approach 2 as approach “*overlap*.”

### Outlier Exclusion Methods

We used ten different methods to exclude outliers: no exclusion, mean **±**2 SD, mean **±**3 SD, quantiles (excluding the outer 5 or 10% of the distribution), *a priori* defined cutoffs, the Tukey outlier criterion, the MAD method (assuming normal distributed data), an adapted MAD method, in which the constant *b* is adjusted according to the Ex-Gaussian distribution and the transformation method suggested by Cousineau and Chartier (2010). As it is difficult to define cutoff values *a priori* given the variety of possible parameters of the simulated distributions, we did not use a fixed upper cutoff for the method using *a priori* defined cutoffs. The upper cutoff was set to the mean RT + 1000 ms. The lower cutoff was set to 200 ms according to the assumption of necessary encoding and response execution time (Ashby and Townsend, 1980; Luce, 1991; Whelan, 2008). Table 1 shows an overview of the outlier exclusion methods, including the information how the thresholds for outlier exclusion were defined.

**TABLE 1:** Overview of the outlier exclusion methods.

Method	Lower threshold	Upper threshold	Additional information
no	-	-	no correction for outliers
cutoff	200 ms	mean + 1000 ms	
2sd	mean – 2 × SD	mean + 2 × SD	
3sd	mean – 3 × SD	mean + 3 × SD	
tukey1.5	q0.25 – 1.5 × IQR	q0.75 + 1.5 × IQR	
q10	q0.05	q0.95	
q05	q0.025	q0.975	
MAD	median – 2.5 × MAD	median + 2.5 × MAD	*b* = 1.4826
MAD_adjusted	median – 2.5 × MAD	median + 2.5 × MAD	*b* = 1.1020
transform	–2	2	z-transformation of square root of uniformized RTs

*Method names are abbreviations. q0.25 denotes the 0.25 sample quantile. IQR represents the interquartile range and MAD the median absolute deviation. For the MAD method, b is calculated assuming normality of data, i.e., *b* = 1/z0.75, while z0.75 depicts the 0.75 quantile of a Normal distribution. For the MAD_adjusted method, b is calculated as follows: *b* = (1/z0.75 + 1/qexp0.75) / 2. qexp denotes the quantile function of an Exponential distribution.*

### Statistical Analyses

For each pair of samples an independent *t*-test was calculated to compare the two conditions. We assumed variance equality, as the same *σ* was used to simulate both conditions^4^. *T*-tests showing a *p*-Value of *p* < 0.05 were considered significant. The proportion of significant *t*-tests given an outlier exclusion method (given the population difference) served as metric for the performance of this method. For estimating the influence of applying an outlier exclusion method on the likelihood to obtain a significant difference in comparison with other factors (effect size, sample size, sample variability), we used a linear regression to predict the *t*-Values of the calculated *t*-tests. The (population) difference, the outlier exclusion method (as factor with reference level “no exclusion”) and the mean SD and trial number averaged across both conditions served as predictors. We do not report *p*-Values, as the number of simulated samples / iterations was manually set to a large value and all *p*-Values consequently were smaller than the common threshold (α = 0.05). Finally, we calculated the bias for each outlier exclusion method. The term “bias” is used in the present work to denote a systematic error introduced by outlier exclusion. The present meaning of this term therefore deviates from statistics, in which it refers to the bias of an estimator. The bias of an exclusion method *m* for a given population difference *diff* was defined here as:

$$b\,i\,a\,s\,\left(m|d\,i\,f\,f\right)=p_{s\,i\,g\,n}\,\left(m|d\,i\,f\,f\right)-p_{s\,i\,g\,n}\,\left(v\,a\,l\,i\,d|d\,i\,f\,f\right)$$

*p*_*sign*_ denotes the proportion of significant *t*-tests, either after excluding outliers according to a specific method (*m*) or in valid RTs (*valid*) for a given population difference (*diff*). A positive bias score indicates a larger proportion of significant differences after outlier exclusion compared to valid RTs, a negative bias score a smaller proportion.

## Results

### Sample Characteristics

The descriptive statistics of the simulated samples show the impact of outliers on the RT distribution. Samples including outliers showed a mean shifted to the right, a larger SD and a considerable larger variability in SDs. Table 2 provides a summary of descriptive statistics for valid RTs and contaminated RTs separately for both outlier simulation approaches. Outliers distorted the sample characteristics, and the influence on the sample variability was especially strong. This distortion was more pronounced when outliers were simulated at the tails of the distribution, which also showed up in the skewness of the RT distributions. The skewness for valid samples was 1.49 for both approaches, while the skewness of contaminated samples was 3.05 for *tails* and 1.98 for *overlap*. The influence of the several exclusion methods on the sample characteristics including skewness is shown in Supplementary Material A.

**TABLE 2:** Statistics of the valid RTs and contaminated RTs in millisecond by the two different approaches to simulate outliers.

Sample	Statistic	Mean	SD	Min	Max
**tails**					
valid RTs	*mean*	600.07	90.71	360.38	873.95
	*SD*	175.57	27.24	64.30	409.49
	*N*	60.02	23.08	20	100
contaminated RTs	*mean*	667.81	101.28	360.38	1060.02
	*SD*	374.86	117.35	78.76	939.19
**overlap**					
valid RTs	*mean*	599.96	90.81	362.97	902.26
	*SD*	175.64	27.26	71.81	427.38
	*N*	60.01	23.11	20	100
contaminated RTs	*mean*	636.28	93.67	362.97	965.34
	*SD*	247.43	45.50	71.81	513.03

*Valid RTs are the simulated samples before introducing outliers, contaminated RTs the samples after introducing outliers. *N* is the same in valid and contaminated RTs and therefore omitted for contaminated RTs. *tails* and *overlap* indicate the respective outlier simulation approach. The distribution of the descriptive statistics *mean*, *SD* and *N* is described by Mean, SD, minimum (Min) and maximum (Max) value. The given statistics are aggregated over iterations and conditions / pairs of samples.*

### Comparison of Outlier Exclusion Methods

The methods differed in the proportion of RTs they excluded / identified as outliers. We will report the average of both outlier simulation approaches (*tails*, *overlap*) for the proportion of excluded RTs, as they (mostly) differed only slightly. Methods with more pronounced differences will be addressed separately in the Discussion when comparing the outlier simulation approaches. For the excluded proportions separately for both outlier simulation approaches, *tails* and *overlap*, please see Supplementary Material A. Naturally, “no exclusion” excluded 0% of the data. The “cutoff” method excluded on average 2.2% of the data, “2sd” excluded on average 5.4%, “3sd” 2.8% and “tukey1.5” 7.9% of the data. “q05” identified 6.8% of the data as outliers, while the average proportion of excluded outliers was 12.0% for “q10.” For these methods, one would naturally expect them to exclude 5% or respectively 10% of the data. Nevertheless, especially in small samples, there can be deviations from these expected values due to rounding issues (e.g., 32 × 0.05 = 1.6; an amount of 1.6 RTs cannot be excluded). The “MAD” method excluded on average 10.9% of the data, while the “MAD_adjusted” method excluded on average 15.0% of the data. The excluded proportion of RTs was 6.0% for the “transformation” method.

Several factors such as effect size, sample size or sample variability determine the likelihood to obtain significant differences, whether they are valid (i.e., true differences) or invalid (i.e., false positive results). In order to estimate the influence of the several outlier exclusion methods on the likelihood to obtain a significant result in comparison to the influence of these other factors, we performed a regression analysis. We predicted the *t*-Values of the tests comparing the two conditions in each simulation iteration with a linear multiple regression by the given size of the population difference, the exclusion method (as factor with reference level “no exclusion”) and the mean SD and sample size of both conditions. Table 3 provides the estimated values of this model. Note that the data of both outlier simulation approaches was merged for estimating this model for the sake of brevity. Separate models for both approaches are shown in the Supplementary Material B. According to the coding of the methods variable, each estimated coefficient of a method depicts how the average *t*-Value of a method exceeds the average *t*-Value of no outlier exclusion. *T*-values decreased with increasing variability and increased with increasing sample size. The given population difference was the strongest predictor (coefficient with the largest *t*-value), while the largest coefficient of the predictors “Outlier Exclusion Methods,” “MAD_adjusted” (*t* = 791.4), indicated an influence nearly as large as that of the predictor sample size (*t* = 800.1). Accordingly, the choice of the outlier exclusion method “MAD_adjusted” compared to no exclusion influenced the probability to reject the Null hypothesis similar to a change of the sample size and more than changing the sample SD.

**TABLE 3:** Results of the model predicting *t*-values.

Coefficient	β	*t*	SE
Intercept	–0.988	–468.0	0.002
given difference	0.033	2342.8	<0.001
Sample SD	–0.002	–462.6	<0.001
Sample N	0.014	800.1	<0.001
***Method (reference = no exclusion):***			
cutoff	0.340	186.9	0.002
3sd	0.406	223.3	0.002
q05	0.415	228.2	0.002
transform	0.679	373.6	0.002
q10	0.688	378.9	0.002
2sd	0.691	380.4	0.002
tukey1.5	0.955	525.7	0.002
MAD	1.171	644.5	0.002
MAD_adjusted	1.438	791.4	0.002

*F(12, 10099987) = 606000; *R*^2^ = 0.419.*

*Method names are abbreviations (see Table 1). *SE* = Standard Error. The model was estimated with the merged data of both outlier simulation approaches.*

In order to assess this variation among outlier exclusion methods, we compared them with regard to their bias, i.e., how the proportion of significant *t*-tests deviated from the proportion of significant *t*-tests in the samples of valid RTs. A positive sign of this bias index indicates a bias towards rejecting the Null hypothesis, a negative sign indicates a bias towards keeping the Null hypothesis compared to the decision based on valid RTs.

**FIGURE 2:**
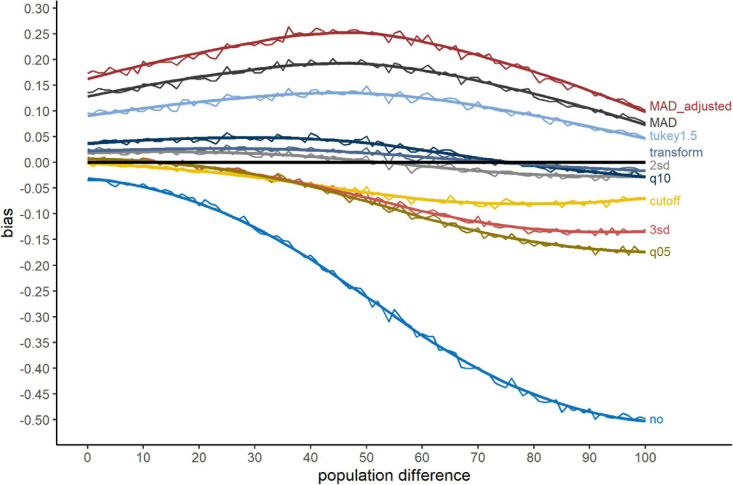
Bias for the outlier simulation approach ***tails***. The *x*-axis shows the population difference between the two conditions. A positive bias value indicates a larger proportion of significant *t*-test after outlier exclusion compared to valid RTs, a negative value a smaller proportion. The black line serves as reference (=no bias). Method names are abbreviations (see Table 1).

Figure 2 shows the bias of the different exclusion methods, when outliers were simulated at the tails of the distribution. The bias ranges from about 25% to -50%. The methods “MAD_adjusted,” “MAD” and “Tukey1.5” show a large Type-I error, i.e., they revealed more significant differences as there were in fact present in the (uncontaminated) data. No outlier exclusion showed a large Type-II error. Especially for large population differences, the presence of outliers concealed actually present differences. The transformation method, “2sd” and “q10” showed the smallest bias, while “cutoff,” “3sd,” and “q05” produced Type-II errors for large population differences. Nevertheless, similar to no outlier exclusion, these methods never provided Type-I errors.

**FIGURE 3:**
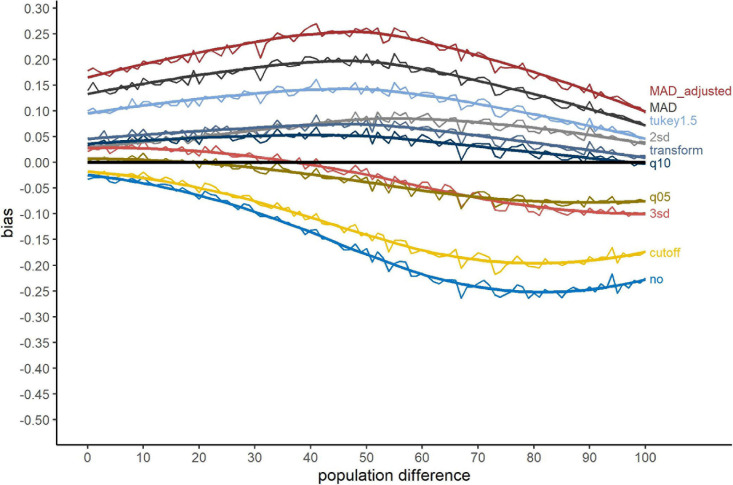
Bias for the outlier simulation approach ***overlap***. The *x*-axis shows the population difference between the two conditions. A positive bias value indicates a larger proportion of significant *t*-test after outlier exclusion compared to valid RTs, a negative value a smaller proportion. The black line serves as reference (=no bias). Method names are abbreviations (see Table 1).

Changing the outlier simulation approach to simulating outliers overlapping with the genuine RT distribution influenced this pattern in several ways. Figure 3 shows the bias when outliers were simulated overlapping with the RT distribution. First, in comparison to Figure 2, the range of the bias decreased. This was clearly a consequence of a reduction of Type-II errors for no outlier exclusion for large population differences from roughly 50% to 25%. For the methods with large Type-I errors, “MAD_adjusted,” “MAD,” and “Tukey1.5,” the pattern remained nearly the same. “2sd” performed worse compared to Figure 2, showing an increased Type-I error rate similar to the “transform” and “q10” methods. “q05” and “3sd” showed smaller biases compared to Figure 2. The “cutoff” method was strongly affected by the different outlier simulation approach with a large increase of Type-II errors.

Table 4 shows the average bias of each exclusion method separately for both outlier simulation approaches as well as the mean bias of both approaches. The less positive the bias score, the lower is the bias in terms of Type-I errors. The less negative the bias score, the lower is the bias in terms of Type-II errors. The lower the absolute value of the bias score, the lower is the average bias of both types of errors. Overall, “q10” showed the smallest absolute bias, while the absolute bias for “2sd” and “transform” was comparable small. The absolute bias was elevated for “3sd,” “q05,” and “cutoff.” Nevertheless, these methods showed negative biases, i.e., only Type-II errors, but no Type-I errors and can therefore be considered conservative. The other exclusion methods showed substantial positive biases, i.e., biases towards Type-I errors. The absence of outlier exclusion (method “no”) showed the largest absolute bias associated with a large number of Type-II errors. Considering differences between the two outlier simulation approaches, *tails* and *overlap*, there were only little. Methods showing a large absolute bias (“no,” the “MAD” methods, “Tukey1.5”), showed those large biases for both approaches. Considering the other methods, there were some minor differences. The methods “2sd,” “cutoff,” “transform,” and “q10” showed larger absolute biases for the simulation approach *overlap*, while “3sd” and “q05” performed worse in terms of absolute bias for the approach *tails*. Nevertheless, the direction of the bias stayed the same and these methods outperformed the absence of outlier exclusion as well as the “MAD” and “Tukey1.5” methods consistent for both approaches.

**TABLE 4:** Average bias of outlier exclusion methods according to the different approaches to simulate outliers.

	Bias		
Method	*mean*	*tails*	*overlap*
no	–**0.212**	–**0.265**	-0.160
MAD_adjusted	0.202	0.202	**0.202**
MAD	0.156	0.155	0.157
tukey1.5	0.110	0.108	0.113
cutoff	–0.087	-0.050	**–**0.124
q05	**–**0.059	**–**0.080	**–**0.038
3sd	**–**0.049	**–**0.068	**–0.030**
transform	0.033	0.012	0.053
2sd	0.031	**–0.001**	0.062
q10	**0.030**	0.023	0.036

*Methods are sorted according to the absolute value of the column *mean*. The less positive the bias score, the lower is the bias in terms of Type-I errors. The less negative the bias score, the lower is the bias in terms of Type-II errors. The largest and smallest absolute values of the bias per column are depicted in bold. *Tails* and *overlap* indicate the respective outlier simulation approach, *mean* depicts the mean of the columns *tails* and *overlap*. Method names are abbreviations (see Table 1).*

To investigate outlier exclusion more general, we also examined the correlations between the bias of the different methods and the proportion of excluded outliers per method. For equally weighting positive (Type-I error) and negative (Type-II error) bias values, we calculated these correlations with the absolute values of the bias. As the “no exclusion” method clearly performed worst and therefore showed a large impact on the magnitude of these correlations, we calculated these correlations without the “no exclusion” method. When outliers were simulated at the tails of the distribution (*r* = 0.662) the association of the proportion of excluded outliers and bias was more pronounced compared to the approach of simulating outliers overlapping with the genuine RT distribution (*r* = 0.446). Nevertheless, for both approaches, excluding a higher proportion of RTs was associated with a larger bias.

## Discussion

The present study compared the bias of several RT outlier exclusion methods using a simulation approach. Bias was defined as the deviation of the proportion of significant differences a method revealed from the proportion of significant differences in the uncontaminated samples. A positive sign of this bias index indicates a bias towards rejecting the Null hypothesis, a negative sign indicates a bias towards keeping the Null hypothesis compared to the decision based on valid RTs. Two different approaches to simulate outliers were used. In the first approach, *tails*, outliers were simulated at the tails of the RT distribution and were consequently always faster or slower than genuine RTs. In contrast, outliers in the second approach, *overlap*, were simulated independent of the genuine RT distribution and could therefore overlap with valid RTs. To briefly summarize the results of the present simulation study, excluding no outliers showed the largest absolute bias, while the exclusion method based on the outer 10% quantiles of the distribution showed the smallest absolute bias averaged across both outlier simulation approaches. Nevertheless, beyond the absolute size of the bias, further points should be considered before recommending a specific outlier exclusion method. These are the type of the resulting error and differences between the two chosen approaches to simulate outliers. The points will be addressed in the following sections before comparing the exclusion methods in more detail.

### Type of Errors Associated With Outlier Exclusion

The bias of the several tested exclusion methods (see Table 4) was negative or positive. While the absolute value of the bias indicates the size of bias, the sign of the bias reflects the type of error a method is associated with. A positive bias indicates a Type-I error, i.e., the method revealed more significant differences than there were in fact present (in the valid samples). Such a method is therefore biased towards erroneously rejecting the Null hypothesis, i.e., indicating an effect in the absence of a population difference. A negative bias in contrast indicates a Type-II error. These methods tend to keep the Null hypothesis, even when significant differences are present in the population. In view of the problem concerning false-positive results in psychology (cf. Simmons et al., 2011), a Type-I error has to be considered more critical compared to a Type-II error. Several methods tended to overestimate the proportion of significant differences, leading to a critical number of Type-I errors. For “Tukey1.5,” “MAD,” and especially “MAD_adjusted,” the errors range from 10% up to about 25% of false-positive results (see Figures 2, 3). Accordingly, these methods cannot be recommended.

### Commonalities and Differences Between the Outlier Simulation Approaches

The chosen outlier simulation approach, i.e., simulating outliers at the tails of the distribution or simulating outliers overlapping with the valid RT distribution, clearly influenced the shape of the contaminated samples compared to valid samples (see Table 2). Nevertheless, the bias showed a similar pattern for both approaches with some more pronounced differences for particular methods, which can be explained by the differential way outliers affect the RT distributions in these simulation approaches. Considering the global differences, the contaminated samples for the approach *tails* were considerably more skewed compared to the approach *overlap*. The skewness for valid samples was 1.49 for both approaches, while the skewness of contaminated samples was 3.05 for *tails* and 1.98 for *overlap*. Consequently, samples were more strongly distorted when outliers were simulated at the tails of the distribution. This could be the reason for the especially large (negative) bias of no outlier exclusion for the approach *tails*. As outlier influences in the *tails* simulations were stronger than in the *overlap* simulations, retaining outliers was associated with a large likelihood of Type-II errors. The more extreme values for long outliers following the approach *tails* may have been the reason for the smaller bias of the “cutoff” method compared to the *overlap* outlier simulation approach. For the “cutoff” method, we defined the upper exclusion threshold as the mean + 1000 ms. Considering the outlier simulation approach *overlap*, a fixed constant of 1000 ms to exclude outliers may have been too large to detect the outliers at the right tail, resulting in a lower proportion of excluded RTs. Furthermore, “2sd” committed more Type-I errors when outliers were simulated overlapping with the genuine RT distribution. As sample variation was smaller for the approach *overlap* compared to *tails*, the exclusion thresholds based on SDs accordingly had to be attenuated following this simulation approach, resulting in a higher proportion of excluded RTs (for the proportion of excluded RTs separately for the outlier simulation approaches see Supplementary Material A). Methods excluding higher proportion of RTs as outliers tended to be biased towards Type-I errors. Overall, the comparison between these simulation approaches suggest that the distribution of the outliers (*tail* or *overlap*) partially determines the bias of the exclusion methods. Despite subtle differences, MAD and Tukey methods consistently showed in both simulation approaches a large positive bias, i.e., a high likelihood of Type-I errors, whereas “2sd,” “3sd,” “q10,” and “transform” methods had consistently lower absolute biases.

### Comparison of Outlier Exclusion Methods

First, and in line with earlier studies (Ulrich and Miller, 1994; Cousineau and Chartier, 2010), one should never use methods, which exclude a large proportion of RTs as outliers. The proportion of excluded outliers correlated positively with the magnitude of bias and the methods excluding a large proportion of outliers (“Tukey1.5,” “MAD,” and “MAD_adjusted”) showed a high rate of Type-I errors (except for “q10,” see below). The effect (the population difference) being simulated in the Gaussian part of the Ex-Gaussian distributions could explain why methods excluding a large proportion at the right tails show large Type-I errors. Methods like “MAD” excluded a large part of the right exponential tail, “normalizing” the distribution, and therefore pronouncing the differences simulated in the Gaussian part of the RT distribution. Note that these methods should not be used nevertheless, as they already show a large bias when there is no difference present (population difference = 0 in Figures 2, 3).

On the other hand, low-biased methods like “2sd,” “q10,” or “transform” more precisely excluded only the simulated outliers exceeding the right tail of the Ex-Gaussian distribution. As empirical RT distributions are not normal distributed (Ratcliff, 1979; Whelan, 2008; Cousineau and Chartier, 2010), they could preserve the empirical (or in this study: simulated) shape of the RT distribution and the impact of this shape on differences between (experimental) conditions revealed by the *t*-tests. Overall, these methods associated with a low absolute bias (“2sd,” “transform,” and “3sd”), excluded a considerable lower number of outliers. This is a consequence of exclusion methods based on standard deviations (/ z-scores), which are strongly influenced by outliers (Miller, 1991), resulting in elevated exclusion thresholds.

Besides the mere proportion of excluded RTs, it has to be considered, at which tail of the distribution methods exclude outliers. The method ‘‘q10’’ excluded a large proportion of RTs as outliers (12%, more than ‘‘Tukey1.5’’ and ‘‘MAD’’), but showed the smallest average bias of all methods across simulation approaches. Due to its symmetrical implementation, this method excluded the same proportion of outliers at the left and right tail of the distribution. Excluding outliers at the right tail accordingly had more adverse effects than excluding outliers at the left tail of the distribution. (‘‘Tukey1.5^5^” and the “MAD” methods had a large impact on the skewness in terms of “normalizing” the distribution by cutting a large part of the right tail. Compare Supplementary Material A). Note at this point that despite the small influence on bias, excluding a large proportion of RTs as outliers (at the left tail) could reduce power due to shrinking the sample size (see Ulrich and Miller, 1994). Although it was associated with the lowest absolute bias, we would not recommend to use the “q10” method. Methods excluding fixed proportions show no flexibility considering the amount of outliers present in the data. Accordingly, there is no chance to not exclude RTs if there are no outliers present in the data. For example, imagine a situation where all RTs are valid, i.e., no outliers are present in the data. The “q10” method would still exclude a high proportion of RTs, and all of them would be valid RTs (Ulrich and Miller, 1994; Cousineau and Chartier, 2010). The same naturally applies to the “q05” method, although to lesser extent.

Furthermore, we would recommend to exclude outliers. The absence of outlier exclusion produced a large proportion of Type-II errors and was therefore associated with a large absolute bias. Even if Type-I errors are more critical, Type-II errors should be minimized as well if possible. Some outlier exclusion methods could reduce the Type-II error rate without introducing Type-I errors. We are aware that there was no method clearly outperforming the other ones, but several methods showing a small bias (“q10,” “2sd,” “transform,” and “3sd”). Given the inflexibility of methods excluding fixed proportions (“q10”), the methods excluding RTs as outliers based on z-scores (“2sd,” “3sd,” and “transform”) were associated with a low (absolute) bias and clearly outperformed the absence of outlier exclusion in terms of absolute bias. Although several exclusion methods based on SDs / z-scores similarly induced only small biases, following Simmons et al. (2011), we emphasize to select the outlier exclusion method in advance of the study in an *a-priori* fashion. Trying different outlier exclusion methods inflates the researcher’s degree of freedoms, therefore increasing the probability of a false-positive finding (Simmons et al., 2011). Furthermore, we would also emphasize researchers in agreement with the conclusions of Ulrich and Miller (1994) to report the proportion of excluded RTs separately for each condition and which proportion were excluded at the left and right tail of the distribution. This should enable the possibility to roughly estimate the influence of the outlier exclusion process.

### Limitations

The validity of the results we report partially depended on the simulation approach we used including the assumptions we made. We sampled RT distributions using an Ex-Gaussian distribution with specific ranges for the parameters *N*, *μ, SD* and *τ*. Nevertheless, it is necessary for a simulation study to specify given properties in advance and the parameter ranges we used were based on former simulation studies (Miller, 1991; Ratcliff, 1993; Whelan, 2008). Furthermore, outliers were simulated by randomly varying the percentage of outliers to generalize the results across different percentages of outliers after aggregation. Former studies used fixed percentages of outliers and varied them in a factorial way (Ratcliff, 1993; Ulrich and Miller, 1994). To the best of our knowledge, evidence about the precise outlier distribution is lacking. We therefore considered all possible proportions being equally likely (therefore we used a Uniform distribution). Of course, other outlier simulation approaches based on more specific distribution assumptions would have also been possible. The usage of *t*-tests to detect the significant differences between simulated RT distributions can be criticized as well. The simulated RT distributions were Ex-Gaussian and consequently skewed. Normality as requirement of a *t*-test therefore was violated. Due to possible different sample sizes in the two conditions (after outlier exclusion), paired *t*-test could not be performed, which would have been the optimal test considering the design used, that is two conditions sampled from two populations only differing in *μ*. Nevertheless, if the implementation of the *t*-tests had influenced the results, such an influence should be comparable for the different outlier exclusion methods, therefore rendering a bias concerning the comparison of the different methods implausible. The results were based on the direct comparison of single RT distributions and not means in different conditions. The reported outcome accordingly represented statistical comparisons on this single sample level. An implementation of a design comparing outlier exclusion methods on the level of multiple samples and their respective means (multiple RT distributions per conditions) would be more complex. It is questionable how strong the impact of such a more complex design on the results would be. How conservative methods exclude outliers should not be affected by the design of the simulation study. The aggregated means per sample should be influenced in a comparable fashion as for the results reported in the present study. Finally, it remains unclear how accurate the chosen approach to simulate outliers was. The approach was adopted from the outlier simulation approach of the HDDM tool (Wiecki et al., 2013) and the approach used by Ulrich and Miller (1994). The assumption of a higher probability of outliers at the right tail of the RT distribution (compared to the left tail) is plausible due to a physiologically caused minimum of RTs at the left tail of the RT distribution (Ashby and Townsend, 1980). Nevertheless, the precise probabilities of short and long outliers as well as the formulas to calculate the outlier values certainly influenced the results of the present simulation study. However, we compared two different approaches to determine outliers, which showed comparable results.

## Conclusion

To summarize, following the results of the present simulation study we would suggest to exclude outliers based on z-scores / SDs. The absence of outlier exclusion showed the largest negative bias, i.e., an increased likelihood of Type-II errors, and should therefore not be recommended. Some methods should not be used as well (“Tukey1.5,” “MAD,” and “MAD_adjusted”), as they produced a large number of Type-I errors. The methods based on SDs / z-scores showed considerable small (absolute) biases, few Type-I errors and excluded only small proportions of RTs. We would further highly recommend to define the outlier exclusion method *a-priori*.

## Data Availability Statement

The datasets presented in this study can be found in online repositories. The names of the repository/repositories and accession number(s) can be found below: https://osf.io/ky8c3/.

## Author Contributions

AB wrote the scripts, analyzed the data, and wrote the first draft of the manuscript. Both authors designed the study, revised the manuscript, and approved the final version.

## Conflict of Interest

The authors declare that the research was conducted in the absence of any commercial or financial relationships that could be construed as a potential conflict of interest.

## Footnotes

1 Note that there is of course research, in which these processes are of interest, e.g., investigating decreasing attention during an experiment. Whether there is a need to exclude outliers has to be decided according to the topic of interest.

2 https://osf.io/ky8c3/

3 As the function to simulate Exponential distributions in R takes the rate instead of τ as input, the reciprocal of the simulated τ was taken (rate = 1 / τ).

4 We assumed the simulated data to represent RTs drawn from the same population (only differing in *μ*) in two conditions. Therefore, the simulated RTs (including outliers) in both conditions were of same size. Nevertheless, due to outlier exclusion, the corrected samples could differ in size. As paired *t*-tests require equal sample sizes in both conditions, we could not perform paired *t*-tests and had to stay with the assumption of variance equality.

5 A possible adjustment of the Tukey’s method for skewed data was for example given by Hubert and Vandervieren (2008). Following their suggested adjustment, they showed the Tukey’s method to exclude a much smaller proportion of the data as outliers for skewed distributions.
