# Supplementary material for: Comparison of Different Response Time Outlier Exclusion Methods: A Simulation Study
Source: Front Psychol. 2021 Jun 14;12:675558. doi: 10.3389/fpsyg.2021.675558 (PMC8238084; doi:10.3389/fpsyg.2021.675558)
Supplement: Supplementary file 1 [file Data_Sheet_1.PDF]

Supplementary Material

Comparison of different response time outlier exclusion methods:

A simulation study

Berger & Kiefer

A: Sample characteristics per exclusion method

Table A.1 provides the sample characteristics after outlier exclusion according to the several exclusion methods for the outlier simulation approach *tails*. Table A.2 provides the same information for the outlier simulation approach *overlap*.

Table A.1: Statistics of the RT samples after outlier exclusion for the outlier simulation approach ***tails***.

Method	Statistic	Mean	SD	Min	Max
no	*mean*	667.81	101.28	360.38	1060.02
	SD	374.86	117.35	78.76	939.19
	N	60.02	23.08	20	100
cutoff	*mean*	606.11	90.38	362.16	893.94
	SD	190.83	29.57	65.75	403.65
	N	57.77	22.20	17.5	100
2sd	*mean*	596.24	91.32	344.92	889.71
	SD	175.22	36.79	60.56	455.03
	N	57.23	22.13	17.5	99
3sd	*mean*	612.35	93.41	347.55	1021.53
	SD	213.48	62.96	63.98	774.67
	N	58.20	22.43	19	99.5
tukey1.5	*mean*	577.21	91.05	333.22	885.97
	SD	137.51	23.67	44.01	324.09
	N	55.27	21.36	15.5	99
q10	*mean*	604.83	93.00	349.12	948.56
	SD	165.49	51.68	46.81	630.71
	N	53.07	20.90	17	90
q05	*mean*	623.25	96.15	352.65	969.40
	SD	222.75	85.05	60.58	780.08
	N	56.04	21.79	18	94
MAD	*mean*	565.32	90.89	328.06	856.00
	SD	121.32	20.33	39.58	276.16
	N	53.47	20.69	14	98
MAD_adjusted	*mean*	552.63	90.53	321.91	839.15
	SD	104.88	18.04	33.78	255.59

	N	50.89	19.68	12.5	93.5
transform	*mean*	598.67	91.53	352.65	900.48
	SD	170.49	33.36	60.58	502.28
	N	56.72	21.88	17.5	98

The distribution of the descriptive statistics *mean*, *SD* and *N* is described by Mean, SD, minimum (Min) and maximum (Max) value. The given statistics are aggregated over iterations and conditions / pairs of samples.

Table A.2: Statistics of the RT samples after outlier exclusion for the outlier simulation approach ***overlap***.

**Method**	**Statistic**	**Mean**	**SD**	**Min**	**Max**
no	*mean*	636.28	93.67	362.97	965.34
	SD	247.43	45.50	71.81	513.03
	N	60.01	23.11	20	100
cutoff	*mean*	628.11	92.77	362.97	944.67
	SD	228.33	38.95	71.81	390.39
	N	59.58	22.95	18	100
2sd	*mean*	586.38	90.88	347.85	891.76
	SD	151.00	24.53	58.79	369.01
	N	56.34	21.74	17.5	98
3sd	*mean*	612.25	94.08	357.48	965.34
	SD	196.28	46.69	58.79	454.14
	N	58.46	22.52	19	100
tukey1.5	*mean*	577.62	91.16	326.99	909.05
	SD	136.51	23.72	45.04	349.52
	N	55.35	21.44	16	98.5
q10	*mean*	602.62	92.24	351.76	919.05
	SD	156.53	35.33	46.68	359.12
	N	53.06	20.93	17	90
q05	*mean*	614.53	92.96	357.19	941.64
	SD	187.79	41.74	55.38	385.35
	N	56.03	21.82	18	94
MAD	*mean*	565.24	90.81	326.99	871.93
	SD	120.35	19.63	35.26	268.74
	N	53.59	20.79	14	98
MAD_adjusted	*mean*	551.92	90.52	317.05	854.10
	SD	104.72	17.64	30.79	234.51
	N	51.14	19.84	13.5	94.5
transform	*mean*	593.73	91.68	349.73	944.67
	SD	157.26	29.73	55.38	433.34
	N	56.12	21.63	17	97.5

The distribution of the descriptive statistics *mean*, *SD* and *N* is described by Mean, SD, minimum (Min) and maximum (Max) value. The given statistics are aggregated over iterations and conditions / pairs of samples.

Table A.3 provides the proportion of as outliers excluded RTs and the skewness of the RT distribution after outlier exclusion per exclusion method for the outlier simulation approach *tails*. Table A.4 provides the same information for the outlier simulation approach *overlap*.

Table A.3: Proportion of excluded RTs (in %) and skewness after outlier exclusion for the outlier simulation approach ***tails***.

Method	% exclude	Skewness
no	0	3.05
cutoff	3.69	1.63
2sd	4.75	1.32
3sd	3.06	1.76
tukey1.5	7.97	0.79
q10	12.04	1.37
q05	6.82	1.94
MAD	10.95	0.64
MAD_adjusted	15.23	0.50
transform	5.54	1.34

Table A.4: Proportion of excluded RTs (in %) and skewness after outlier exclusion for the outlier simulation approach ***overlap***.

Method	% exclude	Skewness
no	0	1.97
cutoff	0.72	1.80
2sd	6.13	1.05
3sd	2.57	1.45
tukey1.5	7.81	0.84
q10	12.04	1.22
q05	6.83	1.51
MAD	10.76	0.68
MAD_adjusted	14.82	0.51
transform	6.46	1.14

B: Regression model separate for both outlier simulation approaches

Table B.1 shows the estimated values of the regression model predicting t-values for the outlier simulation approach *tails*. Table B.2 shows the values of the respective model for the outlier simulation approach *overlap*.

Table B.1: Results of the model predicting t-values for the outlier simulation approach ***tails***.

Coefficient	β	t	SE
Intercept	-0.925	-305.3	0.003
given difference	0.032	1632.2	<0.001
Sample SD	-0.002	-393.7	<0.001
Sample N	0.014	564.7	<0.001
Method (reference = no exclusion):			
q05	0.510	200.3	0.003
3sd	0.521	204.5	0.003
cutoff	0.600	235.6	0.003
2sd	0.748	293.7	0.003
transform	0.785	308.3	0.003
q10	0.829	325.5	0.003
tukey1.5	1.115	437.7	0.003
MAD	1.332	522.9	0.003
MAD_adjusted	1.603	629.2	0.003

$F(12, 5049987) = 305600$; $R^2 = 0.421$

Table B.2: Results of the model predicting t-values for the outlier simulation approach ***overlap***.

Coefficient	β	t	SE
Intercept	-0.240	-59.5	0.004
given difference	0.033	1700.3	<0.001
Sample SD	-0.005	-374.1	<0.001
Sample N	0.014	574.7	<0.001
Method (reference = no exclusion):			
cutoff	0.079	30.9	0.003
3sd	0.291	113.4	0.003
q05	0.319	124.5	0.003
q10	0.548	213.8	0.003
transform	0.572	223.4	0.003
2sd	0.634	247.5	0.003
tukey1.5	0.795	310.5	0.003
MAD	1.010	394.3	0.003
MAD_adjusted	1.273	497.0	0.003

$F(12, 5049987) = 316600$; $R^2 = 0.429$

C: Plot of the bias in relation to effect sizes

Figure C.1 shows the bias in relation to the effect size Cohen's d for the outlier simulation approach *tails*. The effect size d was calculated by: $d = {mean_2 - mean_1} / {((sd_1 + sd_2)/2)}$.

Mean₁ and *mean₂* refers to the respective mean in the uncontaminated samples (in the respective conditions; condition 2 included the simulated effect, i.e.: $\mu_2 = \mu_1 + diff$), sd_1 and sd_2 to the SD in the uncontaminated samples. The effect was systematically varied on the population level. Effect sizes in the simulated samples could therefore differ. The number of samples included in one point of the figure could accordingly strongly differ. As a negative effect size was less likely to be simulated, those estimates are based on less samples. The number of included samples per effect size is therefore depicted by the size of a point.

Figure C.2 shows the respective bias for the outlier simulation approach *overlap*.

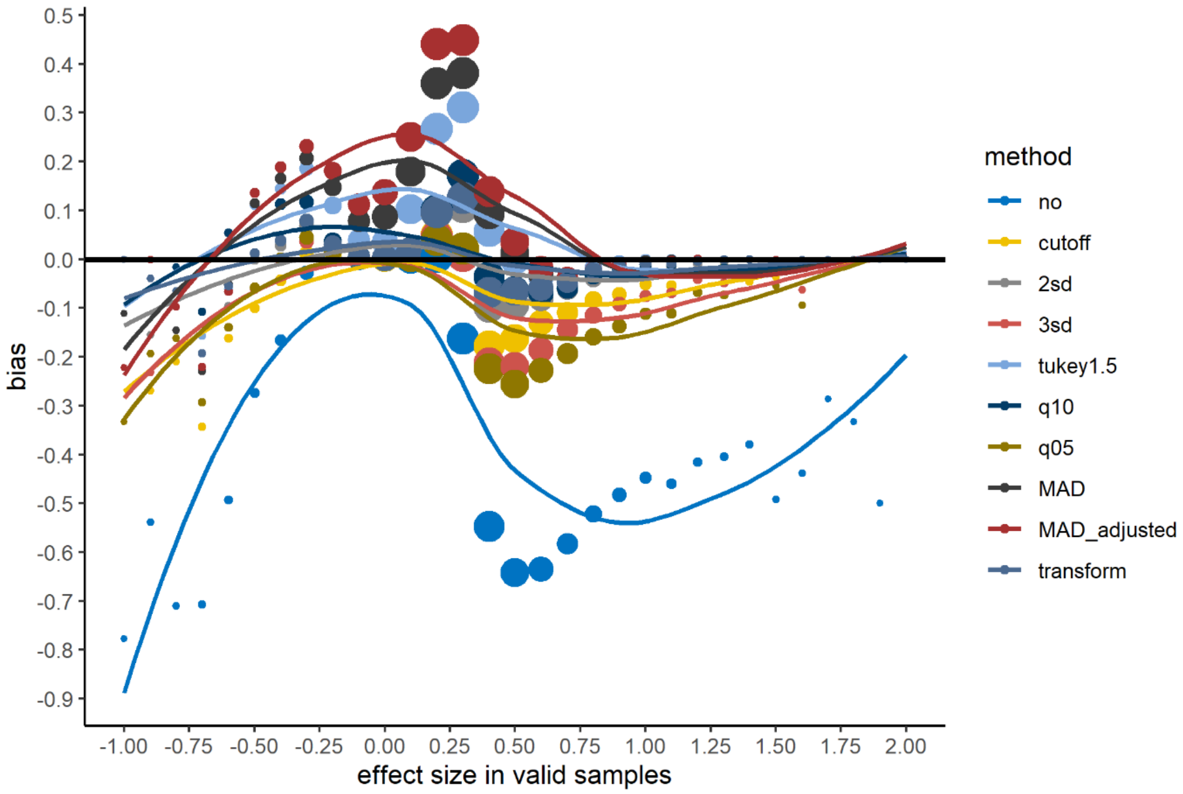

Figure C.1: Bias for the outlier simulation approach ***tails*** in relation to the effect size in uncontaminated samples. The x-axis shows the calculated effect size between the two conditions. A positive bias value indicates a larger proportion of significant t-test after outlier exclusion compared to valid RTs, a negative value a smaller proportion. The black line serves as reference. The size of a point indicates the number of samples included for the calculation of the bias.

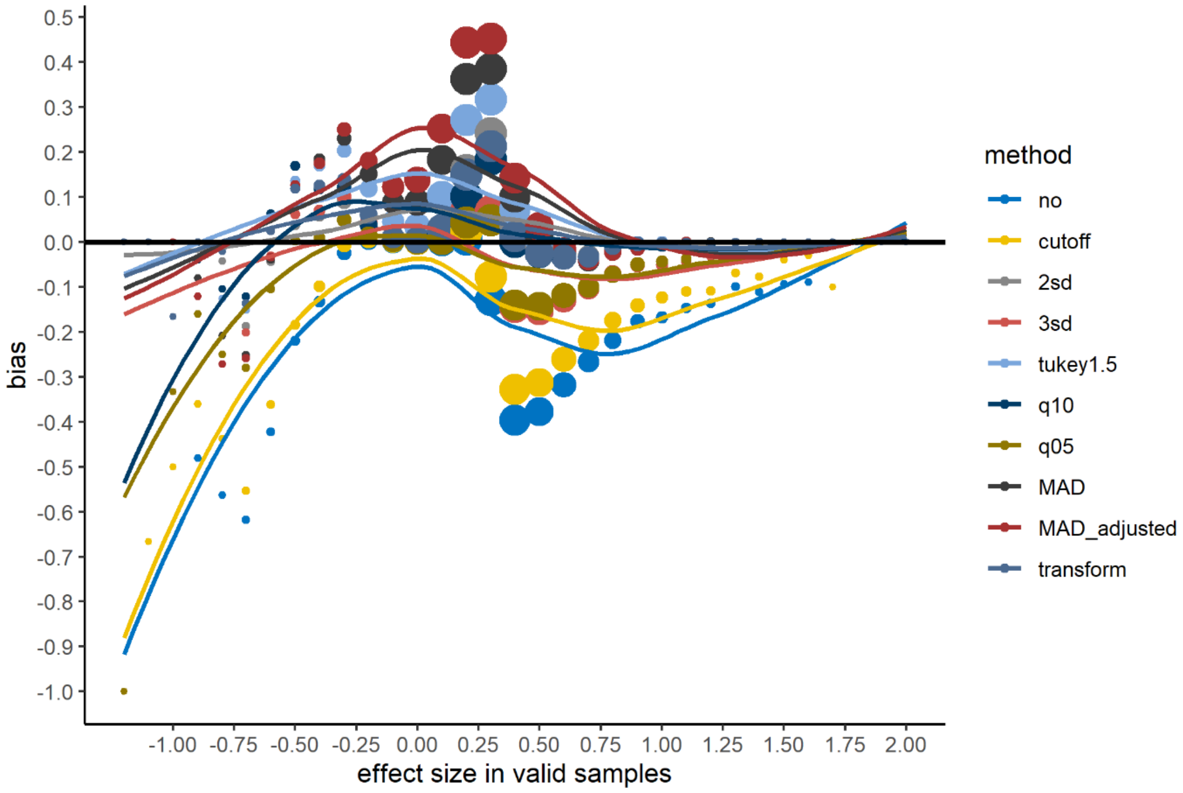

Figure C.2: Bias for the outlier simulation approach ***overlap*** in relation to the effect size in uncontaminated samples. The x-axis shows the calculated effect size between the two conditions. A positive bias value indicates a larger proportion of significant t-test after outlier exclusion compared to valid RTs, a negative value a smaller proportion. The black line serves as reference. The size of a point indicates the number of samples included for the calculation of the bias.
